# Patient-specific 3D-printed nasopharyngeal stents in dogs: a cadaveric pilot study

**DOI:** 10.3389/fvets.2024.1461657

**Published:** 2024-11-20

**Authors:** Craig Sutter, Brian Hardy, Steven Lucero, Lynelle Johnson, William Culp

**Affiliations:** ^1^Veterinary Medical Teaching Hospital, University of California, Davis, Davis, CA, United States; ^2^Department of Medicine and Epidemiology, University of California, Davis, Davis, CA, United States; ^3^Department of Biomedical Engineering, University of California, Davis, Davis, CA, United States; ^4^Department of Surgical and Radiological Sciences, University of California, Davis, Davis, CA, United States

**Keywords:** 3D-printed stents, nasopharynx, nasopharyngeal stenosis, mechanical testing, imperforate nasopharynx

## Abstract

**Background:**

Currently available treatment options for nasopharyngeal stenosis and imperforate nasopharynx in dogs and cats are fraught with complications and failures.

**Objective:**

To develop patient-specific nasopharyngeal stents using 3D-printed molds and to assess placement and fit of stents within the nasopharynx.

**Animals:**

Six canine cadavers.

**Methods:**

Patient-specific nasopharyngeal silicone stents were generated using 3D-printed molds based on CT scans. A placement protocol was developed. Post-placement, goodness of fit within the nasopharynx was evaluated and compared to currently used methods. Mechanical properties of silicone stents were compared to catheter-based and nitinol stents.

**Results:**

Development and placement of stents was successful in all six cadavers. Silicone stents offered stiffness (force required for compression, N) and post-load deformation comparable to nitinol stents (1.8–6.2 vs. 1.2–3.3 N and 0.02–0.08 vs. 0.01–0.14 mm, respectively). Patient-specific stents offered superior goodness of fit in the nasopharynx (81–90%) compared to bilateral red rubber catheters (16.2–33.8%).

**Conclusion:**

Development and placement of patient-specific stents using 3D printed molds was successful in all six cadavers. The novel stents exhibited similar mechanical properties and superior goodness of fit compared to commercially available stents, potentially offering a better alternative to commercially available stents. Further investigation is needed in animals with nasopharyngeal stenosis to determine efficacy and to assess utility in live patients.

## Introduction

Nasopharyngeal stenosis (NPS) and imperforate nasopharynx (INP) are uncommon, yet clinically significant disease processes in the dog and cat. These diseases result in partial or complete occlusion of the nasopharynx leading to decreased, or absent, flow of air through the nasopharynx and subsequently the rest of the respiratory tract. Clinical signs are common and often manifest as mouth breathing, respiratory distress, stertor, gagging, and chronic nasal discharge.

Numerous treatment methods have been described including surgical correction or removal of abnormal tissue, laser ablation, and stenting of the stenotic region (1). Currently, balloon dilation with or without stenting is often recommended as the treatment of choice but suffers from a failure rate reported as high as 50% in cats and 100% in dogs (2). Reported stenting options have included covered or uncovered metallic stents and non-metallic temporary stents (e.g., chest tubes or red rubber catheters) (1, 3, 4). While current stenting techniques tend to result in immediate improvement of clinical signs, they are fraught with short-and long-term complications including infection, foreign body entrapment, stent collapse, stent fracture, stent migration, and the development of oronasal fistulas (1, 2, 5, 6). Stents also frequently have ingrowth of the nasopharyngeal mucosa making their removal extremely difficult without surgical intervention (2). Balloon dilation alone also offers immediate clinical relief, but this procedure carries a high recurrence rate (1). Given the numerous and frequent complications with currently available stents, further research is needed into alternative stenting options.

Recently, literature from human medicine has focused on use of 3D-printing for patient-specific stent development (7–9). These stents have shown promise for providing long-term resolution of obstruction and also offer improved patient comfort (10) and decreased procedural time for placement (11, 12). Additionally, by using a flexible material that can compress and relax with normal physiological respiration, patient-specific silicone stents may provide less complications than routinely used rigid options. The main disadvantage of patient-specific stent use is the time for stent production compared to commercially available stents. In order to minimize production time, in-house production equipment would be required.

With decreasing cost and rising availability of 3D printers, 3D printing is increasingly being utilized in veterinary medicine. Small animal orthopedic surgery is also utilizing this technology to print anatomically accurate 3D models based on computed tomography (CT) imaging that can be used for surgical planning purposes (13, 14). 3D printing is also used routinely for surgical planning in the field of veterinary dentistry and oromaxillofacial surgery (15–17). However, 3D printing for stenting purposes has not been described in veterinary medicine. Given the frequent and severe complications encountered with current therapeutic options for NPS and INP, alternatives to the currently available stent options need to be investigated.

The goals of this study were to describe the development of patient-specific nasopharyngeal stents using a 3D-printed mold, to compare the mechanical properties of this 3D novel stent (3DNS) to currently available self-expanding metallic stents, to define the technique for placement of 3DNS in canine cadavers, and to assess the fit of 3DNS in the nasopharynx.

## Materials and methods

### Animals

Canine cadavers were either purchased from a commercial vendor^1^ or acquired as part of an unrestricted use program at the authors’ institution. Cadaveric specimens were kept frozen prior to evaluation and thawed for 48–72 h at 4°C prior to CT evaluation. Cadavers were chosen from dogs of different sizes (5.7–37.5 kg) in order to evaluate stent development and placement in a range of nasopharyngeal sizes. Three of the dogs were mesocephalic, two were dolicocephalic, and one was brachycephalic.

### Stent production and development

**Figure 1:**
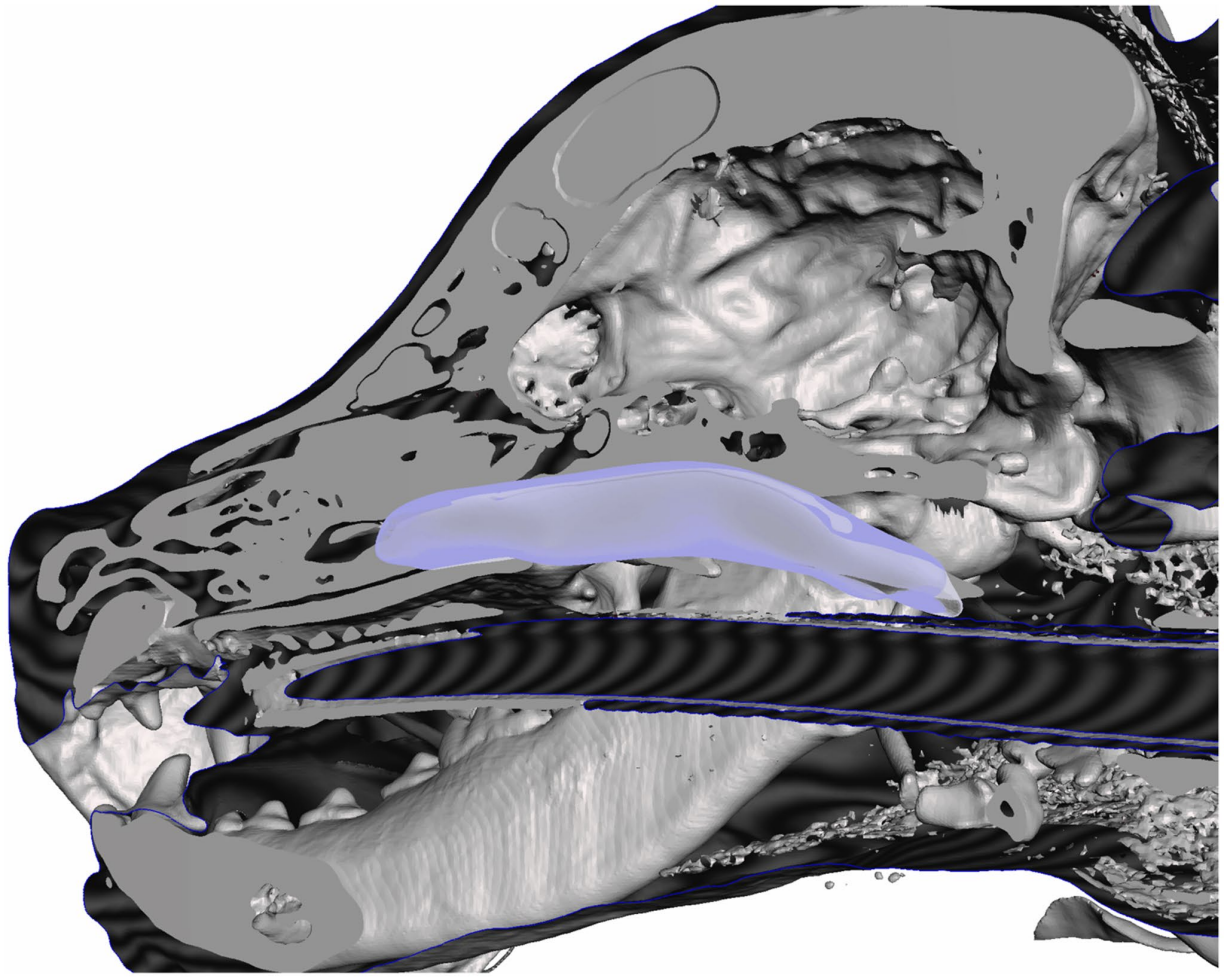
3D reconstruction of a canine cadaver skull outlining the nasopharynx for initial stent prototype development.

**Figure 2:**
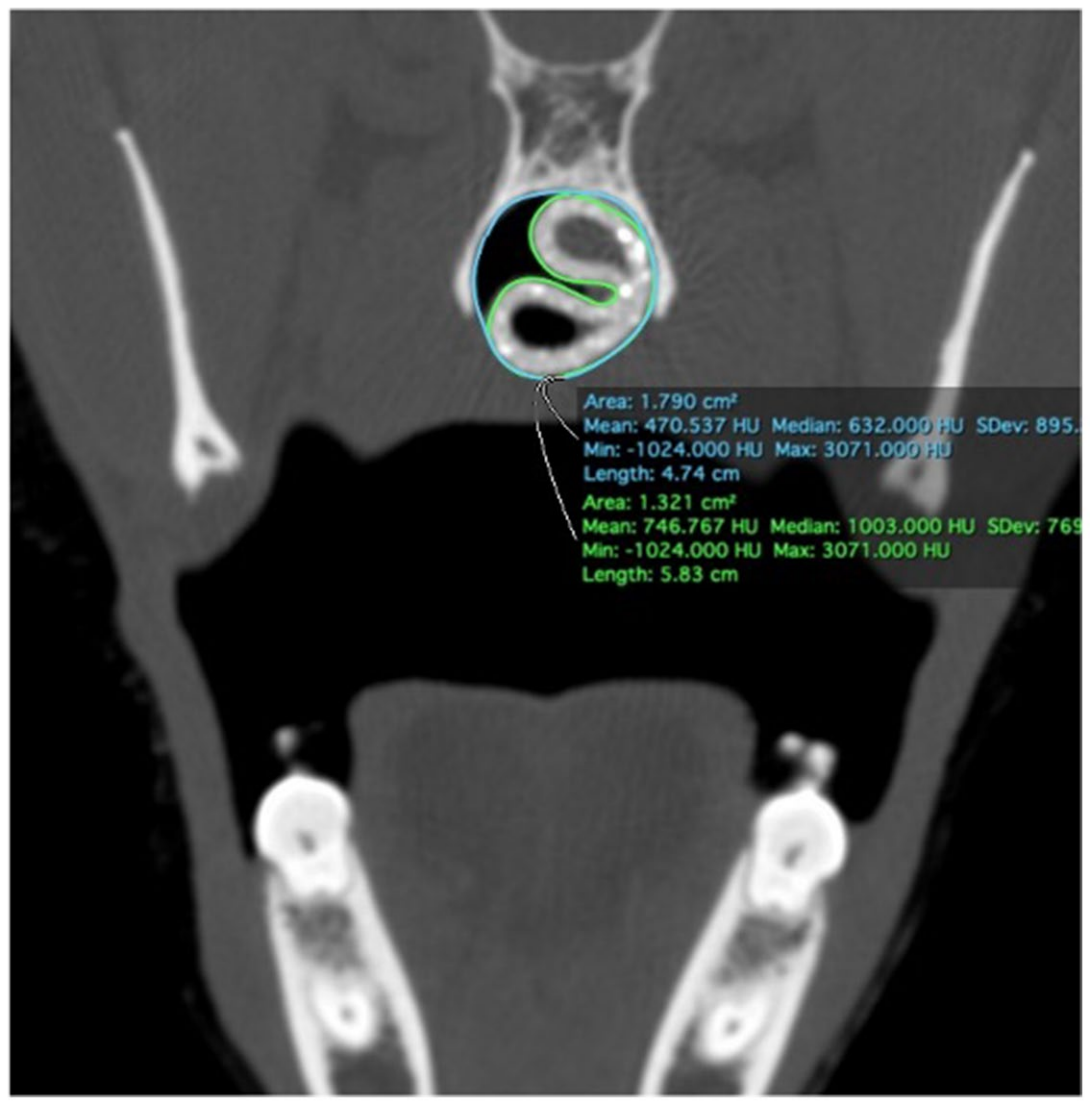
In-folding of 3DNS-4 (outlined in green) post-placement due to oversizing.

**Figure 3:**
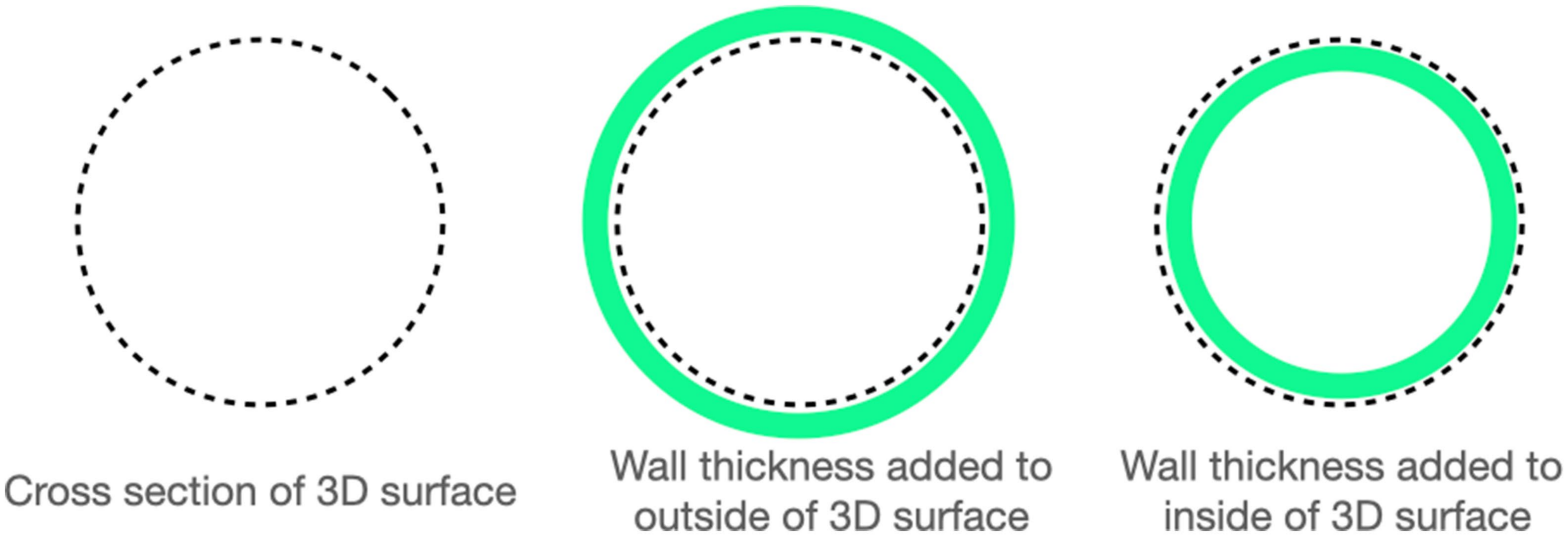
A representation of how wall thickness was determined based on initially determining the cross section of the nasopharynx (far-left) and then adding wall thickness to the outside (middle-image, 3DNS 1 through 3) or to the inside (far-right image, 3DNS4 through 6).

Computed tomographic images of each cadaver head were acquired with open-mouth positioning using a helical 16-slice CT scanner (Lightspeed, 16 helical scanner, General Electric Co., Milwaukee, WI, USA) with 0.625 mm slice thickness. DICOM Images were imported into InVesalius (Center for Information Technology Center Renato Archer, Campinos, Sao Paolo, Brazil) for segmentation. Values between approximately 226–3,071 were used to segment bone and 25–139 for soft tissue from air and to convert the DICOM images into a single standard tessellation language file (.stl). This file was then imported into Autodesk Meshmixer to generate a 3D mesh surface that outlined the nasopharynx from the level of the vomer bone to 75% of the length of the soft palate in its caudal direction (Figure 1). The 3D surface has no thickness, therefore, to generate the stent, the wall thickness was initially added to the outside of the 3D surface for the first three stents (e.g., the 3D surface forms the inner surface of the stent). After observing issues with in-folding of the stents following placement (Figure 2), the protocol was changed to add wall thickness to the inside of the 3D surface (e.g., the 3D surface forms the outer surface of the stent; Figure 3). The wall thickness of the stent was 1.75 mm for cadaver specimens 1, 2, 4 and 5. Wall thickness was reduced to 1.25 mm for dogs 3 and 6 (small breeds) to maintain adequate luminal dimensions.

**Figure 4:**
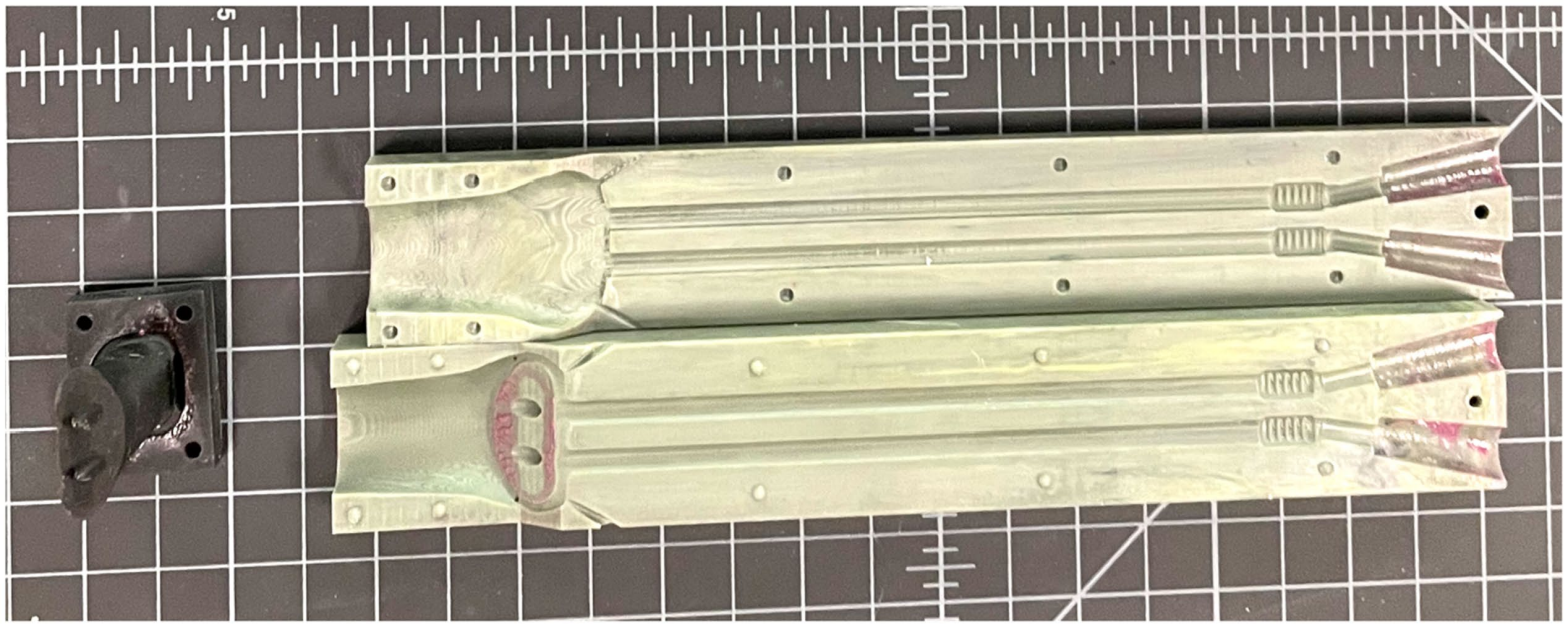
The three-piece 3D-printed patient-specific mold for which the silicone is injected into and subsequently cured.

A 3-piece mold was then 3D-printed with UMA90 resin on a Carbon M2 printer set in standard resolution. The 3 pieces of the mold were comprised of the 2 outer halves, and a third piece suspended in the middle of the mold to form the lumen of the stent (Figure 4). Equal parts of the two-component platinum-cure silicone (Smooth-On Rebound 40) were combined and mixed thoroughly by hand according to manufacturer’s instructions. This mixture was injected using a power injector (Albion Dispensing Solutions) into the mold, held under vacuum at −12 psi for 5–7 min to remove air bubbles, and allowed to cure overnight at ambient pressure.

**Figure 5:**
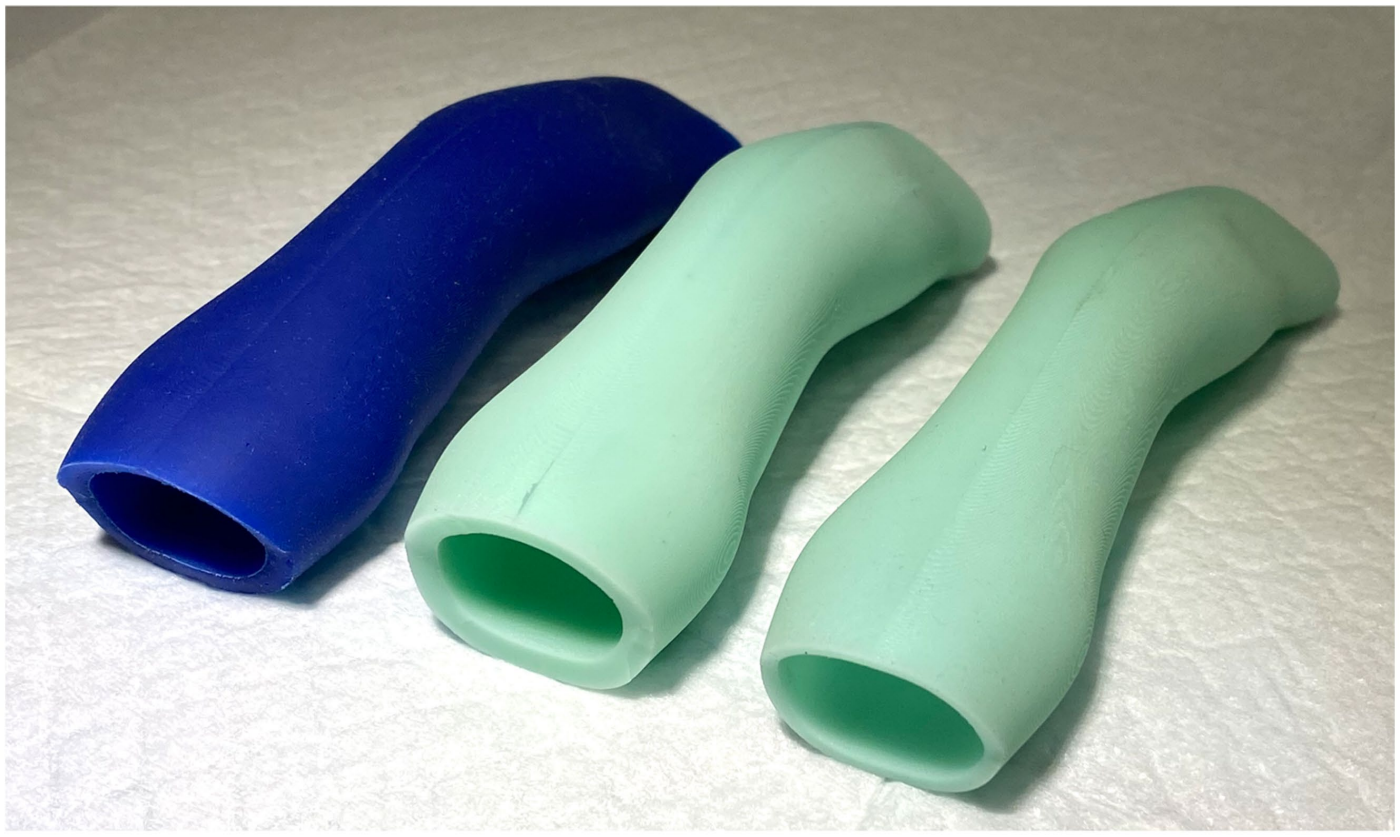
Initial silicone nasopharyngeal stents of varying shore hardness. The left most blue stent was shore 10, the middle stent was shore 60, and the far-right stent was shore 40 and was the ultimate shore hardness used for the 3DNS.

**Figure 6:**
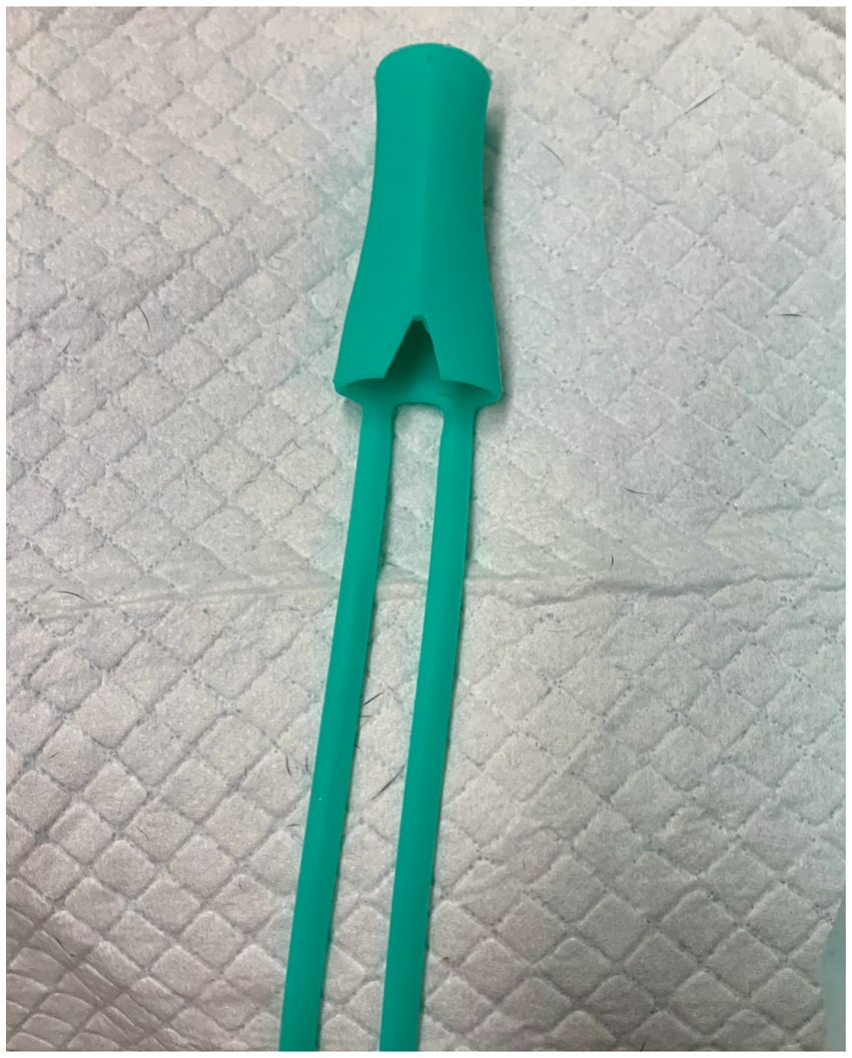
3DNS-1 following wedge shaped removal of rostral-dorsal aspect of stent to accommodate the vomer and prevent stent infolding.

Several prototype stents were created from silicone with different shore hardness and wall thicknesses (Figure 5). Shore hardness is a scale used to measure the hardness of flexible rubbers that ranges on a scale of zero to one hundred with increasing numerical values corresponding to an increase in hardness. Shore A 10 was subjectively deemed to be too soft to resist restenosis, and Shore A 60 was deemed too firm to allow for placement and removal of the stent as well as patient comfort. Shore A 40 was therefore used to produce the 3DNS. The first stent (3DNS-1) was found to fold inward at the level of the vomer on initial placement. 3DNS-1 was removed and a wedge-shaped piece of the dorsal-rostral aspect of the stent was removed to accommodate the vomer and thus alleviate the compression (Figure 6).

**Figure 7:**
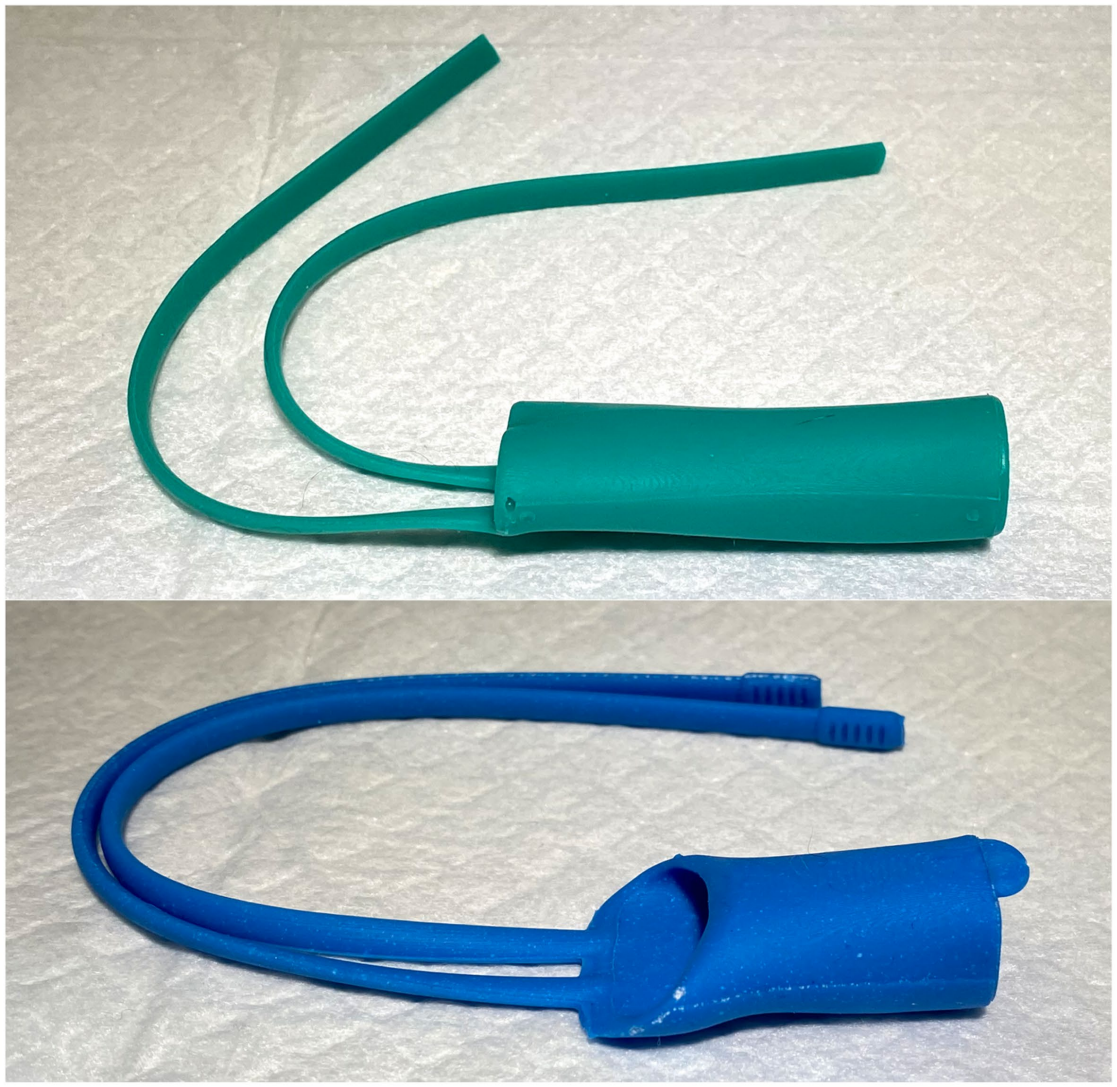
3DNS-1 (top) and 3DNS-2 (below) shows the modifications implemented after placement of 3DNS-1 including thickened and grooved rostral straps, a sloped rostral body to avoid compression by the choana, and a dorsal caudal tab to aid in removal of the stent from the nasopharynx.

3DNS-2 incorporated several changes from experience with 3DNS-1 (Figure 7). The first involved impregnating the silicone with barium sulfate powder to improve C-arm visualization. Specifically, 5 g of barium sulfate powder (98% purity) was added to 100 g of silicone and mixed thoroughly prior to injection into the mold. Secondly, rostral straps were placed to aid in placement and securing of the 3DNS. The tips of the rostral straps were enlarged, and grooves were added to improve traction during placement. Thirdly, a caudal grasping tab was added to facilitate removal of the stent. The final change was creating an angle to the rostral aspect of the 3DNS to match the vomer. This change allowed the stents to be seated rostrally against the vomer but not be collapsed. 3DNS-3 through 6 were produced in a similar fashion.

### Stent placement

**Figure 8:**
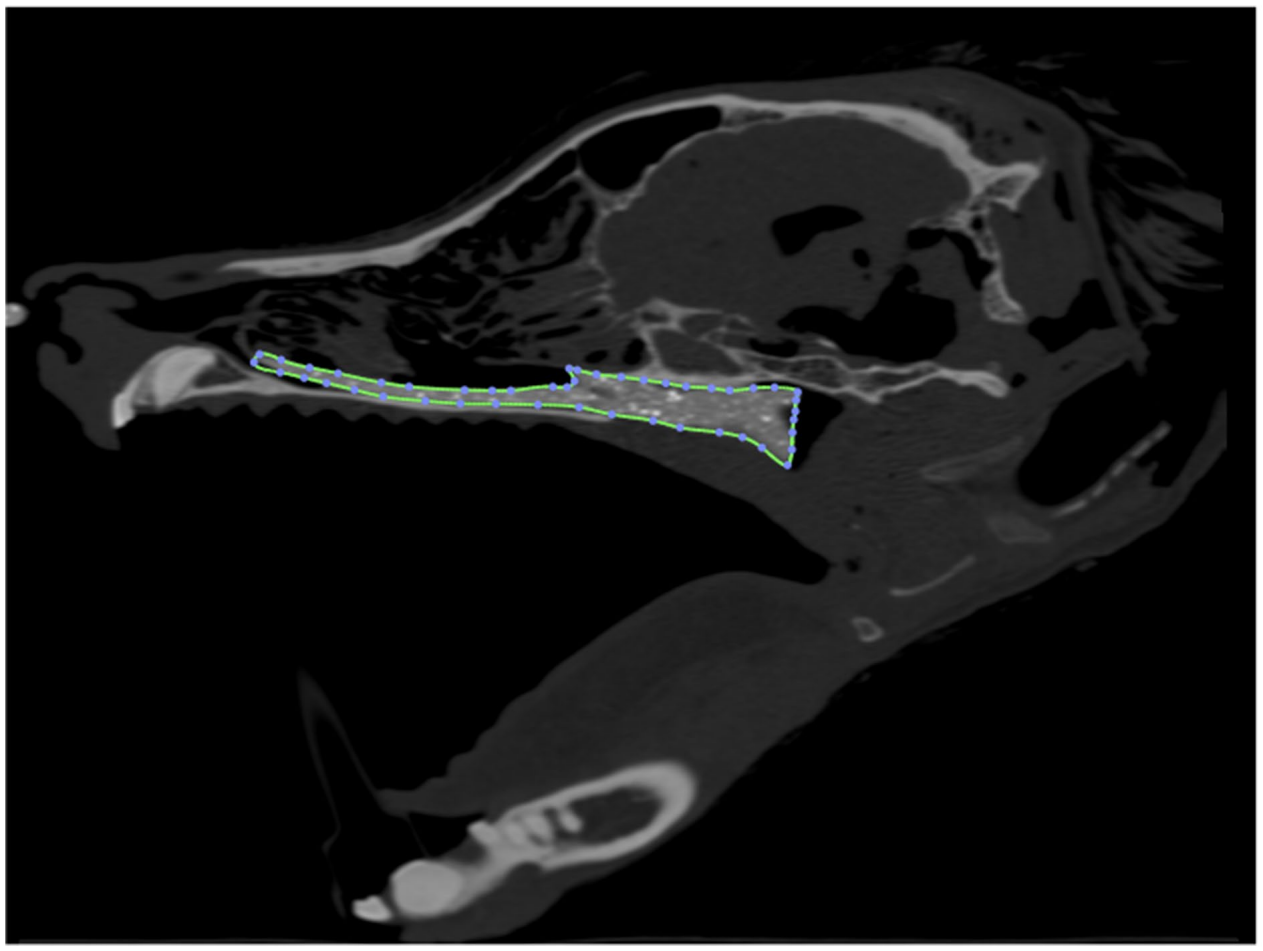
A sagittal CT image depicting 3DNS-3 post placement. 3DNS-3 is outlined for contrast.

**Figure 9:**
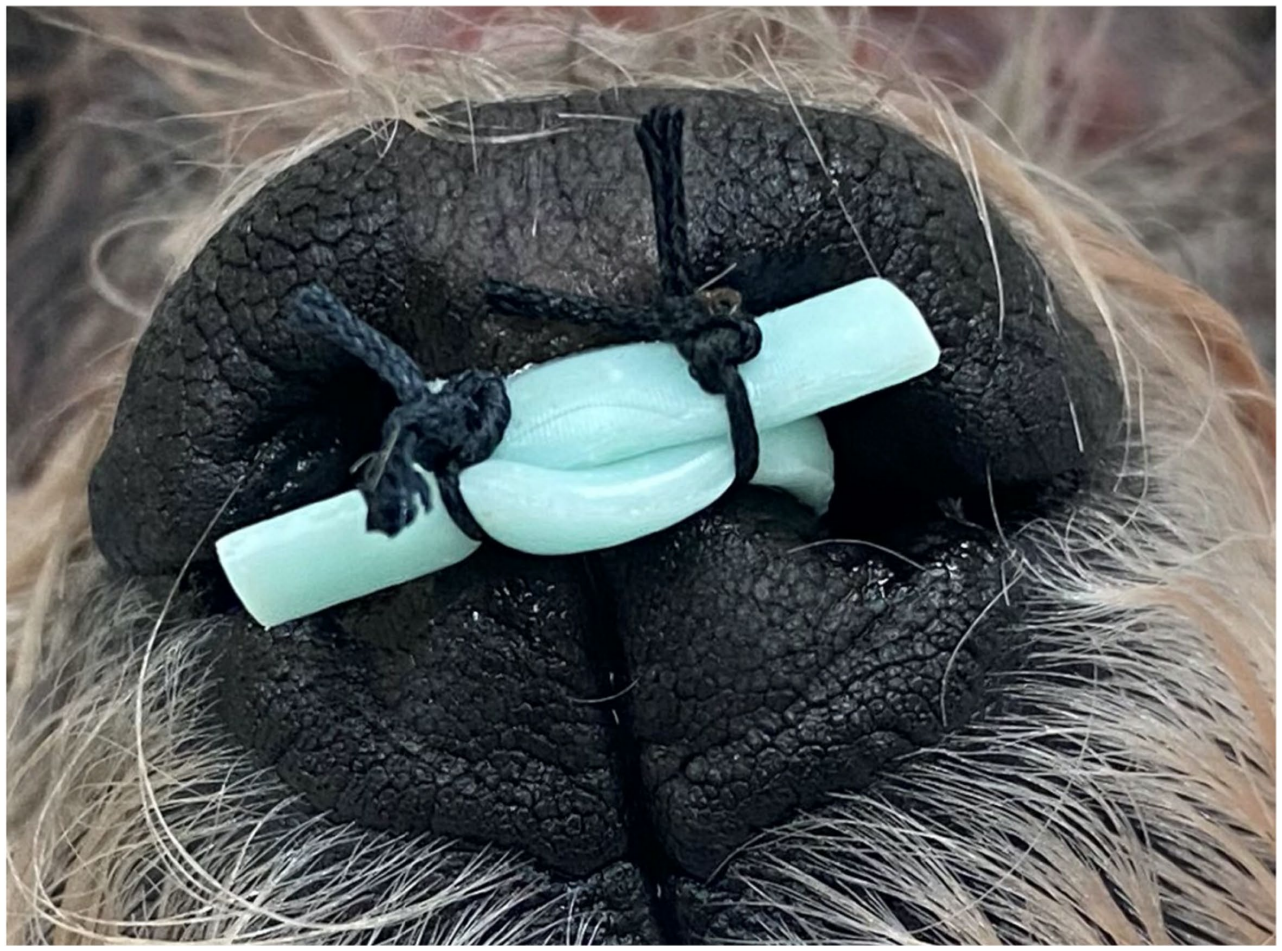
Post-placement image depicting proposed method of securing 3DNS following placement.

The cadaver was placed in right lateral recumbency with a single mouth gag in place on the canine teeth. A flexible videoendoscope^2^ was passed through the oral cavity and retroflexed into the nasopharynx. A 0.035-inch diameter by 150 cm length hydrophilic angle-tipped guidewire^3^ was advanced antegrade through the left naris and ventral nasal meatus under fluoroscopic and endoscopic guidance until visualized in the oral cavity. The guidewire was grasped with a spay-hook and pulled out the mouth. An 8 Fr sheath and dilator^4^ were passed over the guidewire into the nasal cavity. The guidewire and dilator were then removed leaving the sheath in position. This procedure was repeated via the right naris. Flexible endoscopic cup biopsy forceps^5^ were passed bilaterally through the access sheaths to enter the nasopharynx and oral cavity. Once in place, both forceps were used to grasp the straps on the rostral aspect of the stent that was positioned in the oral cavity. The forceps, sheaths, and straps were then retracted under fluoroscopic guidance, and the stent was guided dorsally and rostrally into the nasopharynx (Figure 8). Final position was determined using a combination of nasopharyngoscopy and fluoroscopy to ensure that the stent was firmly seated in the nasopharynx and did not extend beyond the desired region of the soft palate. The straps were then crossed in front of the nasal philtrum to secure the stent in place and 0 silk suture^6^ was used to encircle both straps in two locations; the ends of the straps were trimmed (Figure 9).

### Stent fit assessment

**Figure 10:**
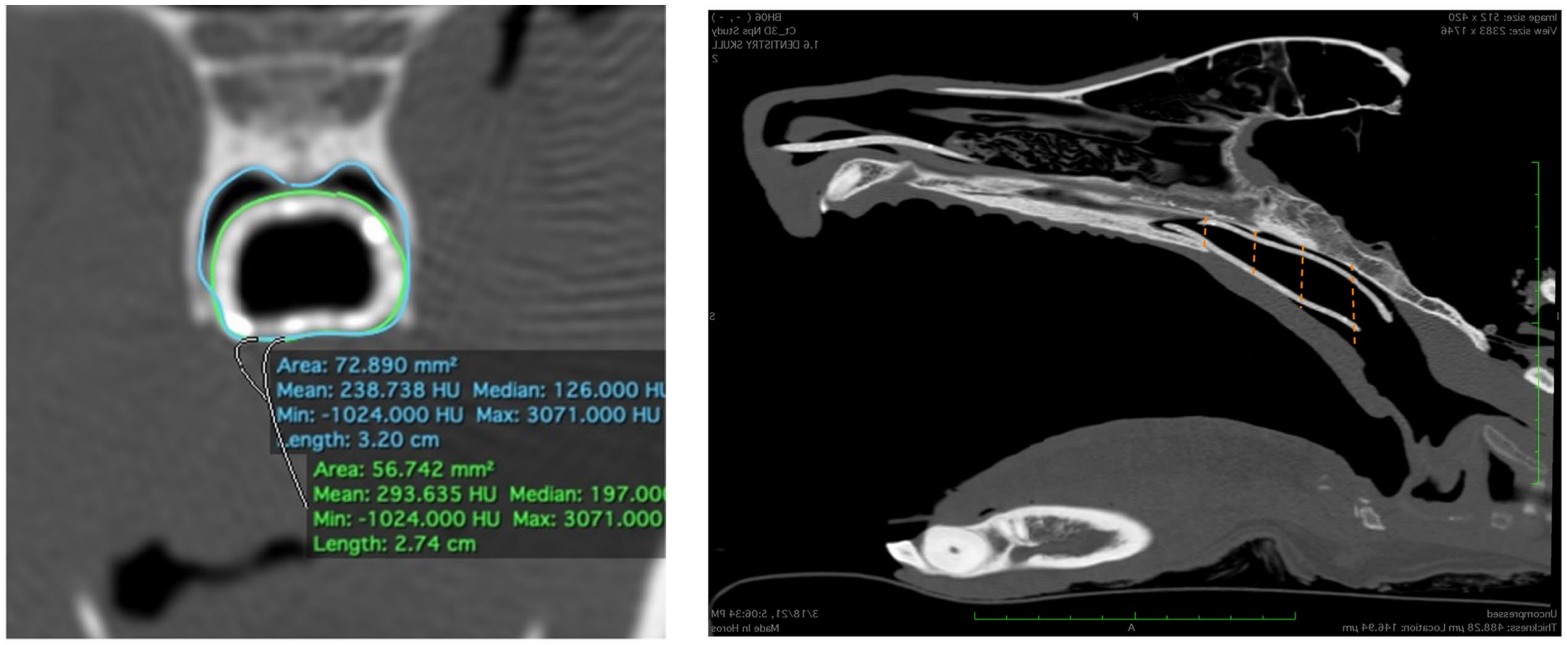
Force-compression curves of all stents (left) and excluding the red rubber catheter and chest tube to show detail of silicone and nitinol stents(right).

Immediately following stent placement, an open-mouth CT scan was repeated for each cadaver. As an objective measure of 3DNS fit, the percentage of the nasopharynx occupied by the stent was calculated on transverse images of the CT scan. Transverse images were acquired at 4 locations over the length of the stent: the rostral and caudal most portions of the entire stent circumference, and 1/3 and 2/3 of the distance between these two points. Cross sectional areas of the nasopharynx and stent were measured with a commercially available software program, (Osirix) using the open polygon function on the equivalent slice at the 4 locations for each cadaver. The percentage area occupied by the stent was calculated for each stent (Figure 10). Two 14 Fr and 18 Fr red rubber catheters were also used with the cadaver from 3DNS-1 to estimate stent fit as red rubber catheters are used at the authors’ institution as removable stents for certain cases.

### Stent removal

Removal was initiated by cutting the silk suture securing the rostral straps of the stent. The flexible videoendoscope was retroflexed into the nasopharynx with flexible biopsy forceps pre-placed through the working channel of the scope. The biopsy forceps were extended to grasp the caudal end of the stent, and a combination of gentle caudal and rotational traction were applied to dislodge and subsequently remove the stent through the oral cavity.

### Mechanical testing

**Figure 11:**
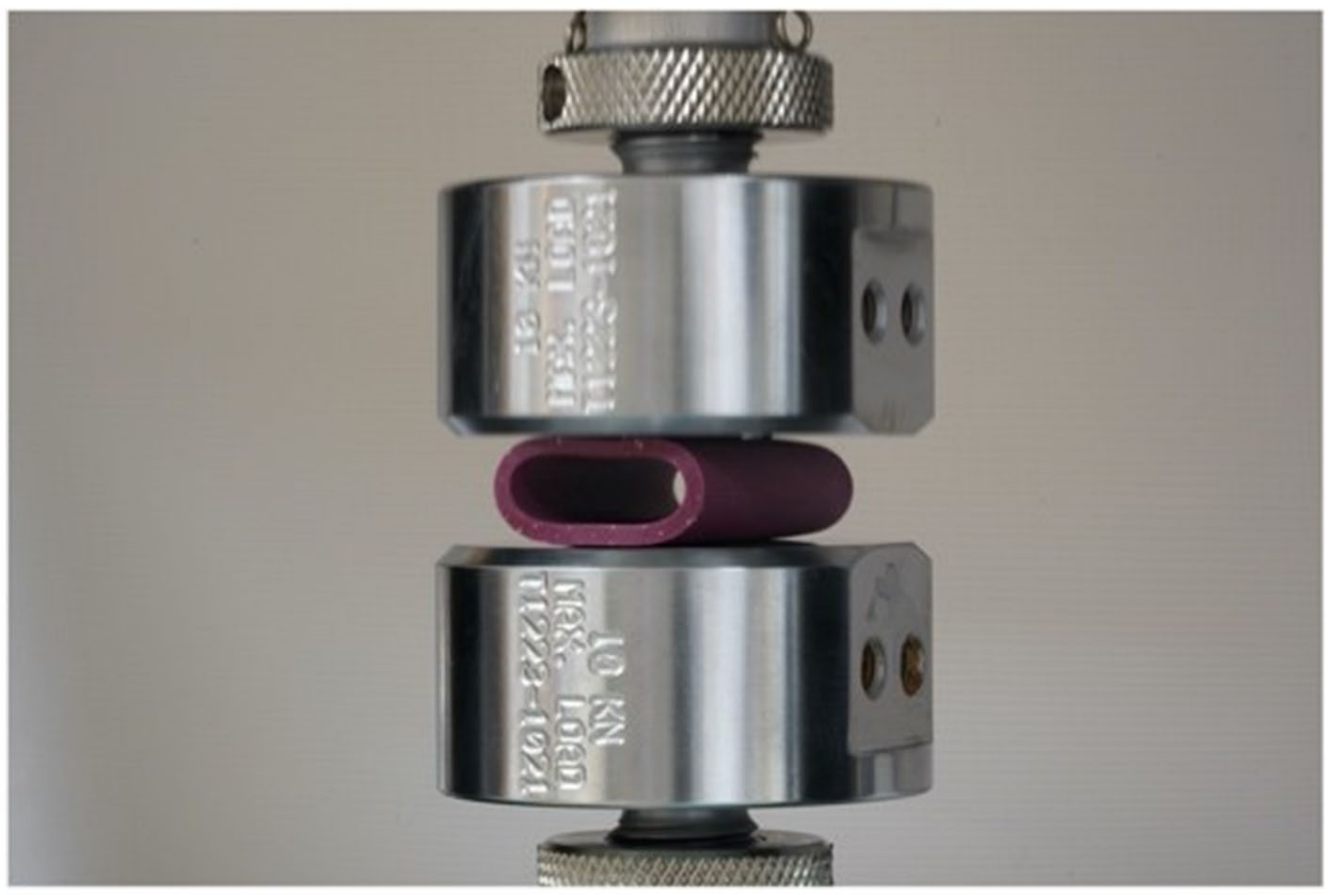
Two compression platens mid-compression of silicone stent during mechanical testing.

Mechanical testing was performed at the UC Davis School of Veterinary Medicine JD Wheat Veterinary Orthopedic Research Laboratory. Due to the asymmetrical shape of the patient-specific stents, cylinders were produced for mechanical testing using identical technique, wall thickness (1.25 or 1.75 mm), and silicone (Shore A 40) as the patient-specific stents. These cylinders were all 40 mm long, with inner diameters of 8 mm (1.25 mm wall thickness), 14 mm (1.75 mm wall thickness), and 20 mm (1.75 mm wall thickness). These diameters were chosen based on the approximate mean diameter of the nasopharynx of the first 3 cadaveric specimens. For comparison, 2 commercially available stents were also tested; a 12 mm × 77 mm woven nitinol stent,^7^ and a 12 mm × 60 mm laser-cut nitinol stent.^8^ A 24 Fr polyvinyl chest tube^9^ (ID 5.72 mm) and an 18 Fr polyvinyl red rubber tube^10^ (ID 3.88 mm) were subjected to the same mechanical testing. The stents were tested on an electromechanical mechanical testing system (Instron model 5,965) with a 1 kN load cell and 4 cm diameter compression platens (Figure 11). Each stent was compressed to 50% of its initial inner diameter at a rate of 12.5 mm/min. This was performed in accordance with standards defined by ASTM International (ASTM 2412). Throughout compression, load and displacement were recorded at 0.05 mm/s. Each stent was unloaded at the same rate as the initial platen position to record residual deformation. Load-deformation curves were subsequently produced and specific parameters collected with custom software (Matlab 2019, Mathworks). Points were captured at a deflection of 5 and 50% inner diameter. At each point of data capture, displacement and force were recorded and energy calculated as the integral under the curve up to that point. At 5% inner-diameter deflection, stiffness was calculated as the least-squares linear fit through all data points up to the point of 5% deflection. Residual deformation was calculated as the difference in displacement at zero-load post-compression relative to pre-load.

## Results

### Stent production, development, and placement

Using individual CT scans, 3DNSs were developed for 6 cadavers. Each prototype was modified to improve stent fit and functionality and to fulfill the first aim of this study. These modifications were previously discussed but include the addition of barium impregnated into the silicone to improve visualization, the addition of rostral straps to aid in securing the stent in place, further modifications to the rostral straps to widen and groove the tips, the addition of a caudal tab to aid in removal of the stent, and lastly, a sloped rostral aspect of the stent to avoid compression by the vomer. All stents were successfully placed and removed with no complications.

### Stent fit

3DNS-1 occupied 76.4–91.6% of the cross-sectional area of the nasopharynx at the 4 sites of measurement along the length of the stent. 3DNS-2 through 6 displayed similar results, ranging from 70.3–95.0% of the nasopharynx occupied by the stent. The 14 Fr red rubber catheters occupied 14.5–16.5% and the 18 Fr occupied 32.8–35.2% of the cross-sectional area of the nasopharynx at the same 4 sites of measurement along the length of the stent. Tables 1, 2 summarize the individual stent results.

**Table 1:** Percent area filled by each individual 3DNS as well as 14 Fr and 18 Fr red rubber catheters (RR).

Measurement location	3DNS1	3DNS2	3DNS3	3DNS4	3DNS5	3DNS6	14 Fr RR	18 Fr RR
Rostral	76.4%	73.8%	90.5%	96.8%	93.3%	86.6%	16.0%	32.8%
1/3 length	85.2%	76.5%	85.0%	95.1%	91.3%	88.0%	16.5%	34.3%
2/3 length	80.7%	85.5%	80.7%	70.3%	85.4%	94.4%	16.3%	35.2%
Caudal	91.6%	91.9%	77.8%	86.6%	85.4%	71.9%	14.5%	33.2%

**Table 2:** Median percent area filled by each individual 3DNS as well as 14 Fr and 18 Fr RR with range.

Median (range)	3DNS1	3DNS2	3DNS3	3DNS4	3DNS5	3DNS6	14 Fr RR	18 Fr RR
	83.0% (76.4–91.6%)	81.0% (73.8–91.9%)	82.8% (77.8–90.5%)	90.9% (70.3–95.1%)	88.4% (85.4–93.2%)	87.3% (71.2–94.4%)	16.2% (14.5–16.5%)	33.7% (32.8–34.4%)

### Mechanical stent testing

Overall, the red rubber catheter and chest tube exhibited the greatest force required for compression with the 24 Fr chest tube requiring 13.77 N for 5% compression and the 18 Fr red rubber catheter requiring 7.11 N for 5% compression. The woven nitinol stent required 0.61 N for 5% compression of inner diameter compared to 0.15 N required by the laser-cut nitinol stent.

**Figure 12:**
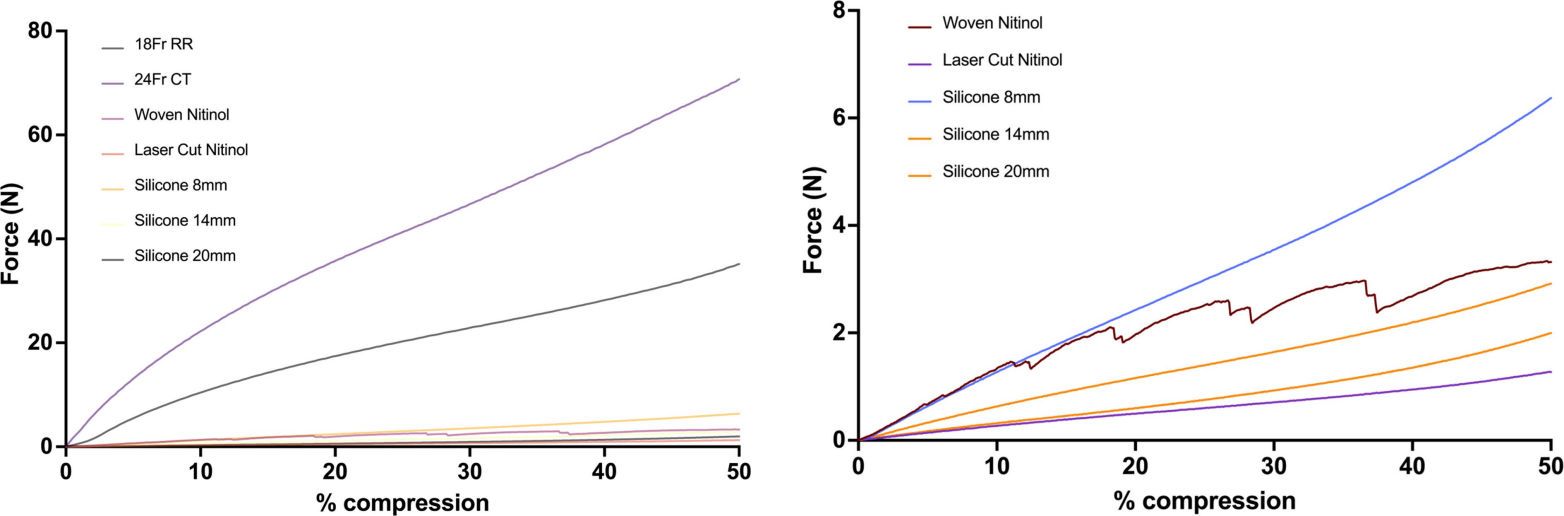
Force-compression curves of all stents (top) and excluding the red rubber catheter and chest tube to show detail of silicone and nitinol stents (bottom).

The 3DNSs displayed a range of stiffness, from 0.17–0.66 N/mm for 5% compression that was inversely proportional to their inner diameter (Figure 12).

**Figure 13:**
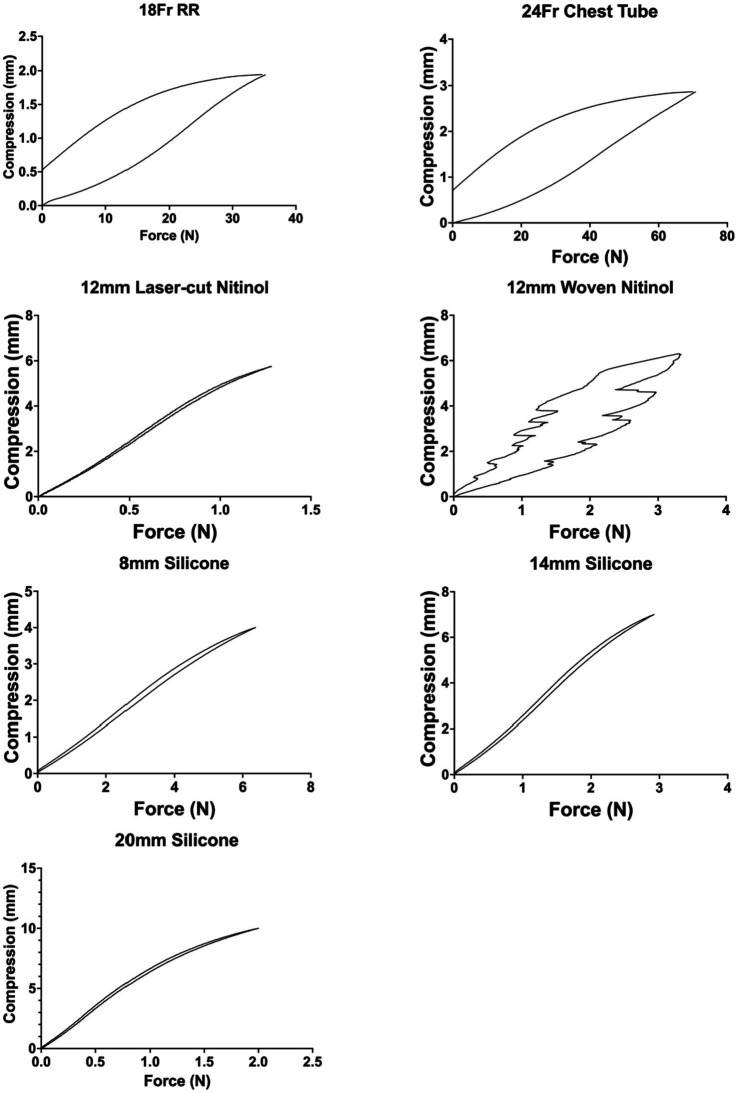
Post-load deformation curves showing compression and relaxation of each stent. The further apart the two lines are, the more post-load deformation has occurred.

Post-load deformation was greatest for the 24 Fr chest tube and 18 Fr red rubber catheter, with 0.73 mm and 0.47 mm of deformation documented, respectively. The woven nitinol stent and laser cut nitinol stent exhibited 0.14 mm and 0.01 mm of deformation, respectively. The 8 mm, 14 mm, and 20 mm silicone stents exhibited 0.02, 0.06, and 0.08 mm of deformation, respectively (Figure 13).

## Discussion

This study describes the successful development and placement of patient-specific nasopharyngeal stents using 3D-printed molds in a range of dog sizes and skull conformations. The stent modifications throughout the process led to improvements in subjective ease of placement and improved fit in the nasopharynx. The procedure itself was relatively easy to perform. The ability to deploy and adjust the stent fit in the nasopharynx allowed for rapid placement and easy adjustments if stent fit was not ideal on first placement. Removal of the stent was also easy, rapid, and minimally invasive whether or not the stent fit well or poorly. Additionally, as 3D printing technology advances, it will likely be possible to directly print flexible, biocompatible patient-specific stents without the need for a mold, eliminating one step from the stent production process described in this study.

There are no clinical trials and only few case reports of patient specific stents created with 3D printing in the human literature. Most of these studies focus on lower airway stenting, however the benefits of patient-specific stents exhibited in those studies provide support for investigating this technique in the upper airway of veterinary patients (7–12). These reported benefits include increased comfort, decreased need for repeated procedures, and improved functionality in restoring patency to a stenotic or malacic airway.

An issue in sizing of the stents was encountered through the development process. In an effort to create an outward force to retain the stent in position within the nasopharynx, as is done for woven and laser cut stents, the wall thickness of initial stents was added to the outside of the outlined nasopharyngeal surface Due to the precise fit of these patient-specific stents, this added enough material to cause in-folding of the stent upon placement because of over-sizing. For the final 2 stents, this thickness was instead added to the inner aspect leading to mucosal apposition without compression.

Mechanical testing of the silicone stents showed comparable stiffness and force required for compression to the commonly used nitinol stents. While the ideal force or stiffness needed to prevent restenosis of the nasopharynx is not known, having comparable results to the nitinol stents suggests the patient-specific stents in this study would likely have the required stiffness needed to prevent restenosis. The custom silicone stents also had less post-load deformation than the red rubber catheters that are currently used at the authors’ institution as stents. Stents with less post-load deformation would be expected to maintain their original shape in a live patient where compressive forces are frequently applied to the stent during respiration and swallowing. Stent fracture has been described in both nitinol stents and silicone stents (18, 19), but further studies are needed to compare the fracture rate between the two stent types.

Ultimately, a clinical trial will be needed to assess the success and potential complications of 3DNS placement in patients with nasopharyngeal stenosis.

Our study had a number of limitations primarily characterized by its proof-of-concept nature and the use of cadavers with normal nasopharyngeal anatomy. Additionally, we are unable to assess complications or success of maintaining patency for these stents. We suspect these stents will carry a similar success in terms of preventing restenosis to commercial stents given their similar mechanical testing. We also suspect they will provide a better cosmetic outcome and superior patient comfort given the lack of mucosal suture required for retention of the stents in the nasopharyngeal region, but future studies will be required to assess this. An additional limitation comes when interpreting our mechanical stent testing, because compression testing requires cylindrical objects and the patient specific stents are not perfectly cylindrical.

One disadvantage held by these proposed stents is the need for multiple anesthetic events. An initial CT scan would be performed to obtain dimensions of the nasopharynx, a second procedure for dilation of the stenosis and placement of the stent once it is produced, and a third for stent removal. Similarly, nitinol stents also require multiple anesthetic events for measurement, placement, and possibly a third event for removal (20). In contrast, placement of modified catheters as temporary stents is the most efficient, with CT scan, dilation and placement of stents performed under the same anesthetic event and removal does not require anesthesia (3).

Overall, this study described the development, modification, and placement of patient specific nasopharyngeal stents using 3D-printed molds. These stents have comparable mechanical properties to that of commercial stents and are developed in a patient-specific method.

## Data Availability

The raw data supporting the conclusions of this article will be made available by the authors, without undue reservation.

## Footnotes

^1^Skulls Unlimited, Oklahoma City, OK, USA.

^2^Olympus BF-P180, San Jose, CA, USA.

^3^Infiniti Medical Weasel Wire 0.035 in × 150 cm; Infiniti Medical, 334 Redwood City, CA, USA.

^4^Arrow percutaneous sheath introducer 8 Fr; Teleflex, Wayne, PA, USA.

^5^Endoscopy Support Services Inc disposable cup forceps; Endoscopy Support Services, Brewster, NY, USA.

^6^Ethicon Perma-Hand Silk Suture; Johnson and Johnson, New Brunswick, NJ, USA.

^7^Infiniti Medical Vet Stent-Trachea; Infiniti Medical, Redwood City, CA, USA.

^8^Infiniti Medical Vet Stent-Urethra; Infiniti Medical, Redwood City, CA, USA.

^9^MILA 24 Fr chest tube; Patterson Veterinary, Loveland, CO, USA.

^10^Patterson Sovereign feeding tube and urethral catheter; Patterson Veterinary, Loveland CO, USA.
